# Optimized Icariin Cubosomes Exhibit Augmented Cytotoxicity against SKOV-3 Ovarian Cancer Cells

**DOI:** 10.3390/pharmaceutics13010020

**Published:** 2020-12-24

**Authors:** Usama A. Fahmy, Omar Fahmy, Nabil A. Alhakamy

**Affiliations:** 1 Department of Pharmaceutics, Faculty of Pharmacy, King Abdulaziz University, Jeddah 21589, Saudi Arabia; nalhakamy@kau.edu.sa; 2 Center of Excellence for Drug Research & Pharmaceutical Industries, King Abdulaziz University, Jeddah 21589, Saudi Arabia; 3 Department of Urology, University Putra Malaysia (UPM), Selangor 43400, Malaysia; docomar82@gmail.com; 4 Department of Urology, University Hospital of Tübingen, Eberhard-Karls University, 72076 Tübingen, Germany

**Keywords:** icariin, cubosome, Box‒Behnken design, apoptosis, G2/M cell cycle arrest, TNF-α down regulation, p53 overexpression

## Abstract

Clinical application of icariin (ICA) is limited, despite its activity against cancer growth, because of the low solubility of ICA in an aqueous environment. Therefore, the present study attempted to develop and optimize ICA-loaded cubosome delivery and to explore its efficacy and possible mechanism of action against ovarian cancer. The optimization of the cubosome formulation was performed using the Box‒Behnken statistical design; during the characterization, the particle sizes were in the range of 73 to 183 nm and the entrapment efficiency was 78.3% to 97.3%. Optimized ICA-loaded cubosomes (ICA-Cubs) exhibited enhanced cytotoxicity and apoptotic potential, compared with ICA-raw, against ovarian cancer cell lines (SKOV-3 and Caov 3). The optimized ICA-Cubs showed a relatively non-cytotoxic effect on normal EA.hy926 endothelial cells. Further analysis of cell cycle arrest suggested a potential role in the pre-G1 and G2/M phases for ICA-Cubs in comparison with ICA-raw. ICA-Cubs increased the generation of reactive oxygen species (ROS) and the overexpression of p53 and caspase-3 in the SKOV-3 cell line. In conclusion, the cubosomal delivery of ICA might provide a prospective approach towards the superior control of ovarian cancer cell growth. Its improved efficacy compared with that of the free drug might be due to the improved solubility and cellular permeability of ICA.

## 1. Introduction

**Figure 1:**
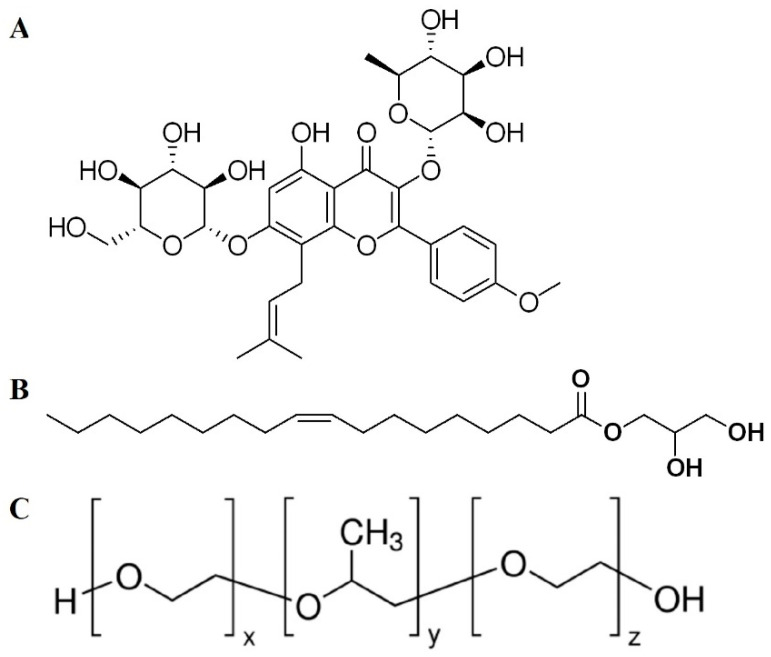
Chemical structure of (**A**) icariin (ICA), (**B**) glyceryl monooleate (GMO), and (**C**) poloxamer 407 (P407).

Of gynecological cancers in women, ovarian cancer is the most common and lethal one, accounting for over 240,000 new cases and 384,000 deaths worldwide in 2018 [1,2]. The 5-year survival rate for ovarian cancers varies from 93% when diagnosed at an early stage to 13.4% when diagnosed at an advanced stage [2]. Even after cytoreductive surgery, chemotherapy cannot improve the survival rate of patients. Recent studies have been focusing on the effective utilization of natural substances in the treatment of malignancies, and of these, the herbal ones are most investigated [3,4,5,6]. Icariin (ICA, Figure 1) is a flavonol glycoside found in plants, most abundantly in *Herba epimedii* (Berberidaceae). Previous reports have investigated the regulatory mechanism of ICA in ovarian cancer [7,8]. The antineoplastic efficacy of this agent has been reported in different malignancies; its efficacy has been shown through the regulation of the proliferation of cancer cells by inhibiting the PI3K/AKT and Raf1/ERK1/2 signaling pathways and promoting apoptosis and cell cycle arrest [9]. It has been shown to regulate the mitochondrial transmembrane potential and caspase-3, with increased release of reactive oxygen species (ROS) within ovarian cancer cells [10]. It has been established that ICA induces apoptosis and suppresses autophagy via the overexpression of autophagy-related p53 in breast cancer cells [11] and that it inhibits the invasion and migration of gastric cancer cells by the vasodilator-stimulated phosphoprotein via the Rac1 pathway [12]. Metastasis and growth of colon cancer was reported to be inhibited by enhancing p53 activity through the regulation of caspase and the imbalance of BCL-2/Bax [13]. In spite of the well-established anticancer potential of ICA, its therapeutic application is limited because of its poor solubility in the aqueous environment, which results in poor bioavailability (~0.1%) [14,15]. Furthermore, it has been established that this agent is metabolized rapidly in vivo via deglycosylation with additional pathways [15]. Nanotechnology-based formulations aid in delivering poorly soluble drugs effectively and safely to the target site [16,17], and researchers have explored different delivery approaches for nutraceutical compounds to improve their pharmacological roles. Such approaches include alginate‒chitosan microspheres [14], poly (ε-caprolactone)/gelatin nanofibers [4], polymeric micelles [18], and phytosomes [10]. Advances in nanotechnology have recently offered a novel approach of delivery for poorly soluble drugs such as cubosomes (Cubs) [19]. Cubs are engineered nanocarriers consisting of bicontinuous cubic phases that possess several advantages for drug delivery. Cubs provide a platform for adjusting the lipids and stabilizers to obtain a carrier with the desired characteristics. Thus, varying the length of the hydrocarbon chain and altering the head-groups of the incorporated lipids can help to obtain the size, charge, and composition of the desired chemical compound, and the stabilizers can also be adapted so they are compatible with and targetable to the site [20]. Accordingly, an attempt was made in this current research to effectively deliver ICA to cancer cells by incorporating the drug within the tunable structure of Cubs, which was stabilized using poloxamer 407 (P407). Glyceryl monooleate (GMO, see Figure 1) is an emulsifier and dispersing agent that forms a film around dispersed globules or lowers the interfacial tension in an emulsion. Structurally, GMO has two parts in its chemical structure, a hydrophilic part and a lipophilic part. They form a protective barrier around a dispersed droplet by adsorbing the oil–water interface, and they stabilize the emulsion by decreasing the interfacial tension of the system. For this reason, GMO was used as an emulsifier in the Cubs preparation [21]. P407 (see Figure 1) block copolymers were introduced in the late 1950s and have been used for many different pharmaceutical applications, including parenteral, inhalation, ophthalmic, and topical formulations. The addition of P407 to the Cubs resulted in an increased complex viscosity of the Cubs forming the solution, indicating the presence of a tighter network that was more resistant to the erosion of the prepared Cubs [22].

Optimization of the formula was performed using Statgraphics software (Statgraphics Technologies, Inc., Warrenton, VA, USA). Seventeen formulas were developed by a three-level Box‒Behnken statistical design. The size of the Cubs was measured, and then the optimized formula was characterized by a transmission electron microscope (TEM) for surface morphology, stability and release were determined. In addition, the cytotoxicity was evaluated on ovarian cancer cells (SKOV-3, Caov-3) and epithelial cells (EA.hy926) as a model for normal cells to evaluate the selectivity of the optimized ICA‒Cubs. Cell cycle analysis and apoptosis studies were carried out to establish the mechanism of ICA-Cubs cytotoxicity. Caspase-3, ROS, nitric oxide (NO), tumor necrosis factor alpha (TNF-α), and P53 were measured to confirm and understand the cytotoxicity of the cellular mechanism.

## 2. Materials and Methods

### 2.1. Materials

ICA with a purity of approximately 99% was received as gift sample from Egypt Mepaco Arab-co, Cairo, Egypt. Glyceryl monooleate (GMO) was donated by the Kerry Group (Limerick, Ireland). P407 and 3-(4,5-dimethylthiazol-2-yl)-2,5-diphenyltetrazolium bromide (MTT) were procured from Sigma-Aldrich Chemie GmbH (Buchs, Switzerland). Roswell Park Memorial Institute (RPMI)-1640, heat-inactivated fetal calf serum (FCS), sodium pyruvate, nonessential amino acids, antibiotics, and bovine insulin, used for cell culture, were also procured from Sigma-Aldrich Chemie GmbH. The rest of the chemicals used in the experiments were of analytical grade.

### 2.2. Preparation of Blanks and ICA-Cubs

Blanks and ICA-Cubs were prepared following the techniques of Nasr et al. [23]. The blank-Cubs, GMO, and P407 were melted by placing them in a water bath maintained at 70 °C. After that, the molten solution was added to a tube containing 10 mL of deionized water maintained at a similar temperature (70 °C). This combination was mixed continuously using a vortex mixer (Boekel, 270100 Tap Dancer-Vortex Mixer, Feasterville, PA, USA).in order to obtain a homogeneous solution. The mixture was then cooled at room temperature and equilibrated for 48 h. The ICA-Cubs was prepared following the above method by dropping the molten mass containing the ICA into the deionized water. The rest of the steps followed were the same as mentioned above.

### 2.3. Optimization of the ICA-Loaded Cubosomal Formulations

A Box‒Behnken statistical design with three factors and three levels (Statgraphics software, version 15.2.05, Statgraphics Technologies, Inc., Warrenton, VA, USA) was used to optimize the ICA-Cubs formulations. Based on the available data in the literature, the maximum and minimum levels of the three independent variables (e.g., the active drug ICA, polymer GMO, and P407) were used for the identification of suitable combinations of the drug and polymers using the Quality by Design technique [23,24]. The software generated 17 different batches of different combinations of the drug, GMO, and P407 at their low (−1), medium (0), and high (+1) levels (Table 1). The 17 batches were developed and characterized for two dependent variables, the particle size and the entrapment efficiency (EE) of the ICA in the formulations, and the data are shown in Table 1. After an analysis of the data of dependent variables using the software, the optimization of the ICA-Cubs was achieved. The effect of the composition of the independent variables on the dependent variables was analyzed using the software, and the optimized formulation was suggested. The analysis of variance (ANOVA) was used to statistically investigate the data to evaluate the significance of the studied variables. Interactions between the independent variables were assessed from the generated contour plots and interaction plots [25]. The best fitting model was selected based on an adequate precision ratio and the predicted and adjusted determination coefficients for the measured response. The equation representing the best fitting model was generated by the software.

### 2.4. Characterization of ICA-Cubs

#### 2.4.1. Determination of Particle Size and the Polydispersity Index and ThermodynamicStability

The dynamic light-scattering technique was incorporated to measure the particle size of the formulated Cubs using a Zetasizer (Malvern Instruments, Ltd., Malvern, UK). After dispersing the ICA-Cubs with deionized water, each sample was measured in three replicates. All the measurements were carried out at a temperature of 25 °C and at a laser wavelength of 633 nm; the scattering angle was 173 degrees, with a medium viscosity of 0.8872 cP and a refractive index of 1.33. The dispersion of the formulated Cubs was prepared by dispersing 0.5 mL of the sample into 30 mL of deionized water to obtain a suitable scattering intensity. The same instrument at the same temperature determined the polydispersity index (PDI) of the prepared formulas. The optimized ICA-Cubs formula was subjected to three freeze‒thaw cycles at −20 °C (12 h) and +25 °C (12 h). Each formula then was inspected for particle size.

#### 2.4.2. Transmission Electron Microscope Examination

Morphological evaluation of the formulated and optimized ICA-Cubs was done with the TEM (Jeol Company, Tokyo, Japan) equipped with a Super Twin lens. This experiment was carried out by placing a drop of the cubosomal dispersion on a 200-mesh, carbon-coated copper grid. The drained sample was stained using 1% sodium phosphotungstate solution and allowed to dry for 15 min at 25 ± 1 °C. 

#### 2.4.3. Estimation of Entrapment Efficiency 

The Entrapment Efficiency (EE) of the ICA within the ICA-Cubs was determined by the centrifugation method of Nasr et al. [24]. Briefly, 1 mL of the prepared ICA-Cubs was added to 4 mL of deionized water and centrifuged at 15,000 rpm for 15 min until complete precipitation of the cubosomal nanoparticles was achieved. One milliliter of the clear supernatant was added to 4 mL of methanol, and the mixture was vortexed for 5 min. A glass tube was filled with 100 µL of the vortexed solution, 20 µL of HCl 36% solution, and 2 mL of acetonitrile. The mixture was mixed well for 1 min and centrifuged at 5000 rpm for 20 min. Thereafter, the supernatant was evaporated in a clean glass tube. Finally, reconstitution of the residue was done using the mobile phase (200 µL) and then injected in high-performance liquid chromatography (HPLC) for analysis of the ICA [26]. The EE (%) of the ICA-Cubs was calculated using the following formula (Equation (1)):

(1) $$EE\;=\;\frac{Amount\;of\;drug\;entrapped}{Total\;amount\;of\;drug}\;\times 100$$

#### 2.4.4. Determination of Release Profile of ICA-Cubs

In vitro release of the ICA-Cubs and the ICA-raw was performed using the cellulose tube diffusion technique. The cellulose tube was soaked in the release media overnight before starting the experiment. The cellulose tube was filled with an accurately weighed amount of the Cubs, equivalent to 100 mg of ICA. After being tightly closed, it was immersed in a receptor compartment containing 900 mL of phosphate buffer (pH 7.4). The release of the ICA was performed at 37 ± 0.5 °C using the U.S. Pharmacopoeia dissolution apparatus (paddle method) at 100 rpm with 900 mL of phosphate buffer as the dissolution medium [27,28]. Two milliliters of the released media was collected from the dissolution vessels at predetermined time points of 0.5, 1.0, 2.0, 4.0, 6.0, 8.0, 12.0, and 24.0 h, followed by replacement with an equal volume of fresh media at the same temperature to maintain the sink conditions. All the collected released samples were filtered using a 0.45-µm syringe filter prior to analysis by HPLC [26]. These analyses were performed in triplicate, and the mean value was considered for further evaluation.

### 2.5. Evaluation of Cytotoxic Effects of Optimized ICA-Cubs on Ovarian Cancer Cells

#### 2.5.1. Cell Culture and Maintenance

The human ovarian cancer cells SKOV-3 and Caov-3 in our current experiment were obtained from the American Type Culture Collection (Manassas, VA, USA). While and EA.hy926 cells were obtained from National center for cell science (NCCS), Pune, India. The cell lines are presently available in the cell bank of the Tissue Culture Unit and maintained in an RPMI-1640 medium, supplemented with FCS (10%, *v*/*v*), bovine insulin (1 µL/mL), streptomycin (100 µg/mL, *w*/*v*), penicillin (100 U/mL, *v*/*v*), and sodium pyruvate (1 mM) in a humidified environment at 37 °C and 5% carbon dioxide (CO_2_).

#### 2.5.2. Cytotoxicity Study

Cytotoxic evaluation of the ICA-Cubs was done in the ovarian cancer cell lines following the 3-(4,5-Dimethylthiazol-2-yl)-2,5-diphenyltetrazolium bromide (MTT) assay. In order to accomplish the experimental objective, 3 × 10^3^ cells of SKOV-3 and Caov-3 were seeded onto each well of 96-well plates, followed by incubation at 37 °C in a 5% CO_2_ incubator for 24 h. Different concentrations (0.1 µM to 1000 µM) of ICA-raw, ICA-Cubs, and staurosporine as a control were incubated with the cancer cells for 24 h. Finally, the MTT assay was performed following reported methods [29,30], in which the incubated cells were treated with MTT solution (0.5%, *w*/*v*) for 4 h after the removal of traces of samples. Later, dimethyl sulfoxide was used to dissolve the formed formazan crystals, and the optical density of the formazan solution was measured using the enzyme-linked immunosorbent assay (ELISA) microplate reader at 490 nm. The noncancerous EA.hy926 endothelial cells were cultured in Dulbecco’s modified Eagle’s medium (DMEM) and were then treated in the same way as mentioned for the ovarian cancer cells. 

#### 2.5.3. Cell Cycle Analysis

Flow cytometry analysis of the SKOV-3 cells was performed to determine the cell cycle arrest by the formulations [31]. Here, 1 × 10^5^ cells were seeded into the well plate with the ICA-Cubs, blank-Cubs, ICA-raw, and staurosporine as a positive control at a concentration of 5 µM for all treatments. Following 24 h of incubation, the cells were harvested with 70% ethanol overnight at 20 °C. In the next step, propidium iodide/RNAse staining solution (at 5 µg/mL each) was used to stain the cells following incubation for 3 h in the dark. Finally, the stages of cells in different cell cycles were determined using a Cytek^®^ Northern Lights 2000 spectral flow cytometer (Cytek Biosciences, Fremont, CA, USA), and the acquired data were analyzed by using SpectroFlo™ Software version 2.2.0.3 (Cytek Biosciences, Fremont, CA, USA).

#### 2.5.4. Determination of Apoptotic Potential

The apoptotic potential of the formulated ICA-Cubs was compared with that of the ICA-raw and blank-Cubs by the dual staining technique following the published method [10,32]. During this analysis, 1 × 10^5^ numbers of SKOV-3 cells were incubated in each well of a six-well plate with the concentration of the test material equivalent to 10 µM of ICA, wherever applicable. The staining of the cancer cells was performed by the commercially available Annexin V-APC/propidium iodide (PI) detection kit (BD Biosciences, San Jose, CA, USA). Thus, the detection of early-stage apoptosis was carried out with the (Annexin V plus-PI‒) and the detection of late-stage apoptosis was determined with the (Annexin V plus-PI+), using the flow cytometer FACSCalibur (BD Biosciences). Obtained data were analyzed using Multicycle software (Phoenix Flow Systems, San Diego, CA, USA).

#### 2.5.5. Measurement of Reactive Oxygen Species Production

Determination of the overall ROS in ovarian cancer by the treatment of ICA-Cubs, ICA-raw, and blank-Cubs was carried out in SKOV-3 cells using the Reactive Oxygen Species Assay Kit (BD Biosciences). The cells were grown in a 96-well plate as described in our previous experiment and incubated with the samples for 24 h. According to the manufacturer’s protocol for the kit, the treated cells were further incubated for an additional 30 min in the dark following the addition of 10 µM of 2,7-dichloro-dihydro-fluorescein diacetate. Later, the cells were rinsed with DMEM and analyzed in a flow cytometer to measure the fluorescence intensity. The excitation and emission wavelengths for the measurements of fluorescence intensity were set at 485 nm and 530 nm, respectively, in the instrument [10,33]. 

#### 2.5.6. Measurement of Nitric Oxide Production

This assay of NO production was carried out using the Nitrate/Nitrite Colorimetric Assay Kit (Cayman Chemical, Ann Arbor, MI, USA) following the manufacturer’s instructions. Following incubation of the ICA-Cubs, ICA-raw, and blank-Cubs samples with the SKOV-3 cells in a well plate, the medium was treated with the kit reagent. Absorbance of the mixture was determined in the ELISA microplate reader at 550 nm following 10 min of incubation.

#### 2.5.7. Determination of TNF-α

In this experiment, the SKOV-3 cells were incubated at 37 °C with the ICA-Cubs, ICA-raw, and blank-Cubs with TNF-α for 24 h. After incubation, the cells were detached using 0.25% trypsin and then incubated with 10% flow cytometry buffer (FCB) containing phosphate-buffered saline (PBS) for 30 min at 4 °C following centrifugation. This prevented the nonspecific binding of antibodies. Finally, the extracellular staining of TNF-αR1 was performed, as described in the literature [34]. Briefly, the cells were treated as per the manufacturer’s instructions using phycoerythrin (PE)-labeled-TNF-αR1 antibodies at 4 °C. Following 30 min of incubation, the cells were analyzed by flow cytometry (Becton Dickinson, San Jose, CA, USA). 

#### 2.5.8. Analysis of Caspase-3

Analysis of caspase-3 was performed following overnight incubation of the SKOV-3 cells (5 × 10^4^ cells per well) at 37 °C with ICA-Cubs, ICA-raw, and blank-Cubs samples using the commercially available kit (BD Biosciences). After incubation of the 5 × 10^4^ cells per well, the cells were lysed using the lysis buffer provided in the kit. Then the contents of the wells in the plate were assayed in an ELISA microplate reader at 405 nm [10,33].

#### 2.5.9. Enzyme-Linked Immunosorbent Assay for p53

Analysis of the p53 protein was performed using the ELISA method, where the SKOV-3 cells (3 × 10^3^ cells/mL) were placed in the supplied 96-well plate (Sigma Aldrich, St. Louis, MO, USA). The cells were treated with the ICA-Cubs, ICA-raw, and blank-Cubs samples for 24 h. Thereafter, the wells were incubated with detection antibody and treated with horseradish peroxidase-labeled anti-rabbit Immunoglobulin G (IgG) antibody after washing at each stage. Finally, stabilized chromogen was added to the wells for stabilizing the chromogen that had developed, and a stop solution was added to stop the reaction before taking the reading at 450 nm using the ELISA plate reader.

### 2.6. Statistical Interpretations

Findings of the three samples of each data point were averaged to show the data as the mean ± SD, and the statistical analysis of the displayed results was performed using SPSS software version 25 (SPSS Inc., Chicago, IL, USA). Comparison of the mean data for multiple groups was carried out by the ANOVA followed by Tukey’s post hoc test, where a *p*-value of less than 0.05 indicated statistical significance.

## 3. Results

### 3.1. Optimization of the ICA-Cubs Using the Box‒Behnken Design

#### 3.1.1. Particle Size of the ICA-Cubs

The particle size of nanocarriers plays an important role in cancer therapy because enhanced permeability and intracellular delivery [30]. Thus, an analysis of the particle size based on the independent variables was performed in this study. The statistical outcome of the interactions of the concentrations of the three independent variables, ICA, GMO, and P407, on the particle size is shown in Table 2. The statistical significance of the influence of the ICA (A), GMO (B), and P407 (C) concentrations on the particle size is indicated by the *p*-values (*p* < 0.05) in Table 3. From the obtained analysis, it is clear that the model terms A, B, C, and BB are significant because the *p*-values of those models are less than 0.05.

Furthermore, the R-squared value was 98.9923%, whereas the adjusted R-squared value was 97.1786%. 

A polynomial equation was generated based on the interactions of the independent variables and the effect on the particle size of the ICA-Cubs formulations (Equation (2)). For the particle size response, the regression coefficients for ICA and GMO are recorded as +0.562163 and +329.326, respectively, and the coefficient of P407 is –0.220801.

Y1 (Particle size) = 195.361 + 0.562163A + 329.326B − 0.220801C + 0.00186667AA + 0.7AB − 0.00148936AC + 304.167BB − 0.478723BC + 0.00301796CC(2)

**Figure 2:**
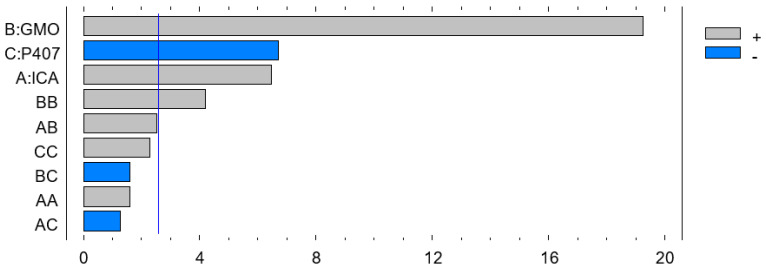
Pareto chart showing the effect of the ICA, GMO, and P407 concentrations on the particle size of the ICA-loaded cubosomes (ICA-Cubs) formulation.

The Pareto chart on the effect of the independent variables on particle size is shown in Figure 2. Therefore, the coefficient in Equation (2) and the Pareto chart are in agreement, where the positive effect of ICA and GMO is evident in both Equation (2) and on the Pareto chart (see Figure 2). The increased size with increasing ICA and GMO concentrations might be explained by the increased content of GMO in the developed Cubs. Our findings are in agreement with those in the reported literature [23,24]. As per the Pareto chart (see Figure 2), the GMO concentration has the greatest effect on the particle size, and this is also supported by the highest coefficient of independent variable B in Equation (2). 

**Figure 3:**
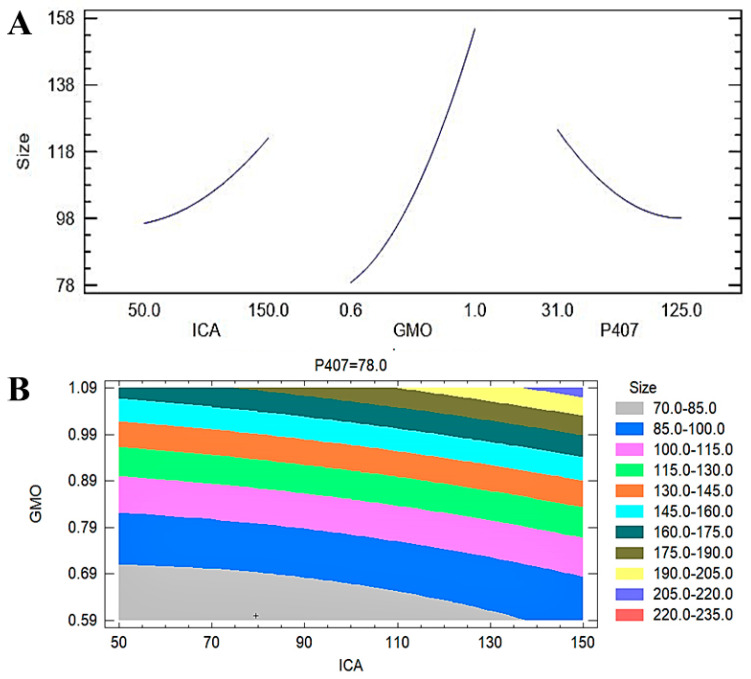
(**A**) Main plot effect of ICA, GMO, and P407 concentrations on the EE of ICA-Cubs. (**B**) Contour plot of the effect of ICA, GMO, and P407 concentrations on the particle size of ICA-Cubs.

A contour plot (Figure 3B) further confirmed the observations in the Pareto chart and the main plot effect on the particle size of the ICA-loaded cubosomal formulations. The colors in the contour plot clearly show the maximum changes in the particle size from 70 to 85 nm to approximately 160 to 175 nm with the changes in the GMO concentration. The changes in particle size with the changes in the ICA concentration are limited to a lower range. The particle size is in the higher ranges on the P407 axis. The surface charges of the developed 17 ICA-Cubs were found to be within the range of +21.25 ± 0.345 mV to +28.25 ± 0.362 mV. Our findings on the surface charges of the formulated cubosomes are in agreement with those in the literature [24]. The PDI is used to see whether the colloidal system has a homogeneous size; the PDI was found to be 0.35 to 0.13. The findings indicate good thermodynamic stability of the optimized formulas, with no significant differences in particle size after and before the three freeze‒thaw cycles.

#### 3.1.2. Entrapment Efficiency of ICA-Cubs

The EE is considered one of the important physicochemical characteristics of pharmaceutical nanoformulations. It indicates the quantity of drug incorporated within the developed formulation [35]. In this study, we used the centrifugation method to determine the EE of the ICA within the formulated ICA-Cubs. The statistical outcome of the interactions of the three independent variables on the EE is shown in Table 3. The statistical significance of the influence of the ICA (A), GMO (B), and P407 (C) concentrations on the EE of the ICA-Cubs is indicated by the *p*-values (*p* < 0.05) in Table 2. In Table 3, it is clear that the model terms B and C are significant because their *p*-values are 0.05.

The R-squared value is 96.80%, whereas the adjusted R-squared value is 91.03%. The actual (experimental) and predicted (fitted) values for the EE of the ICA-Cubs are in close agreement, as shown in Table 3, and the percentage errors for the EE values are very low. A polynomial equation was generated based on the interactions of the independent variables and the effect on the EE of the ICA-Cubs (Equation (3)).

Y_2_ (EE) = 50.0927 + 0.0169078*A + 36.0988*B + 0.345694*C − 0.000316667*A^2 + 0.0775*A*B + 0.000223404*A*C − 19.1667*B^2 ‒ 0.0159574*B*C − 0.00118455*C^2 (3)

**Figure 4:**
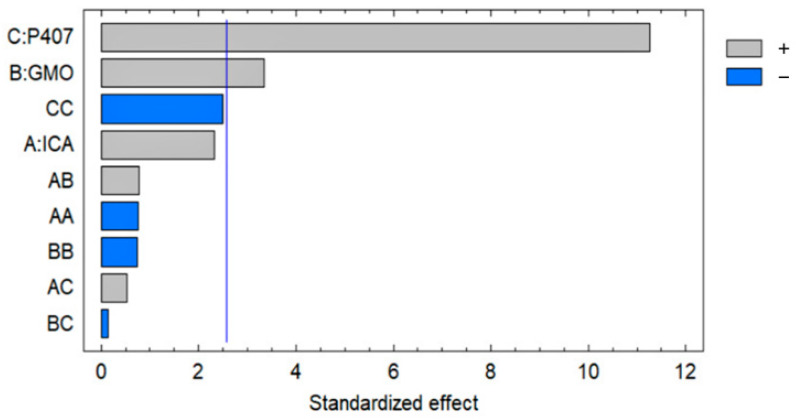
Pareto chart showing the effect of the ICA, GMO, and P407 concentrations on the EE of the ICA-Cubs formulation.

**Figure 5:**
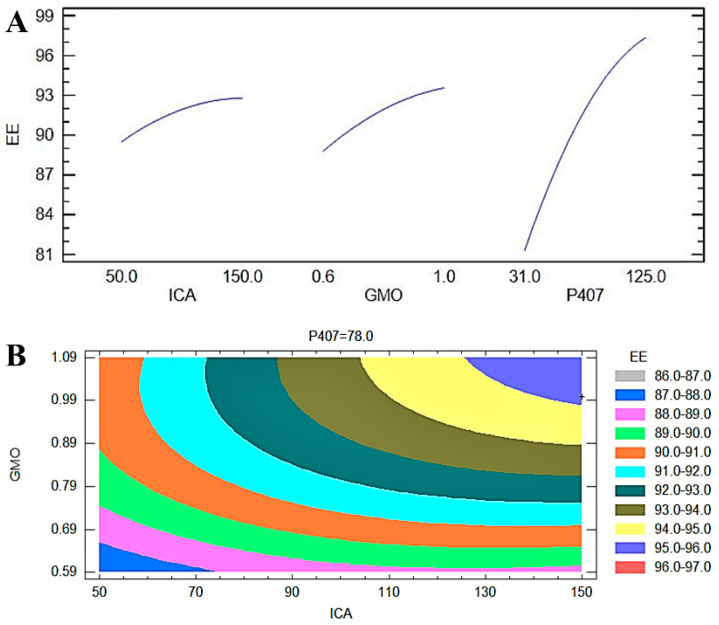
(**A**) Main plot effect of the ICA, GMO, and P407 concentrations on the EE of the ICA-Cubs formulation. (**B**) Contour plot of the effect of the ICA, GMO, and P407 concentrations on the EE of the ICA-Cubs formulation.

The most significant effect of P407 is evident in Figure 3, Figure 4 and Figure 5. A sharp increase in the EE was observed when the P407 concentration was increased, as is evident in the main plot effect in Figure 5). There is a slow increase in the EE shown in Figure 5A with the increase in the ICA and GMO concentrations; however, the effect of the GMO concentration is more prominent than the effect of the ICA concentration. This is reflected in both the Pareto chart (see Figure 5A) and the color changes in the contour plot (see Figure 5B). 

### 3.2. Characterization of the ICA-Loaded Cubosomal Formulations

#### 3.2.1. Interpretation of the Transmission Electron Microscope Analysis

**Figure 6:**
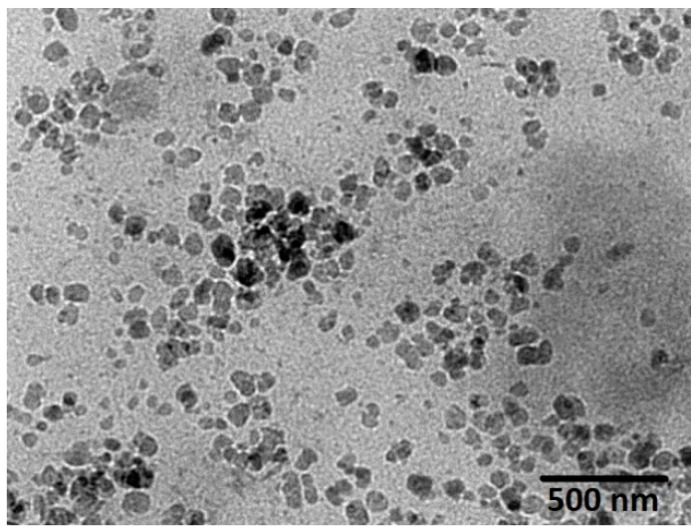
TEM photomicrograph of the optimized ICA-Cubs.

The size of the cubosomal dispersions determined using the Zetasizer instrument was further confirmed by TEM analysis. Figure 6 shows that the optimized ICA-Cubs with irregular hexagonal and polyangular shapes were well dispersed as individual particles. The particle size observed is similar to the findings by Nasr and team [23]. 

#### 3.2.2. In Vitro Release Profile of ICA

**Figure 7:**
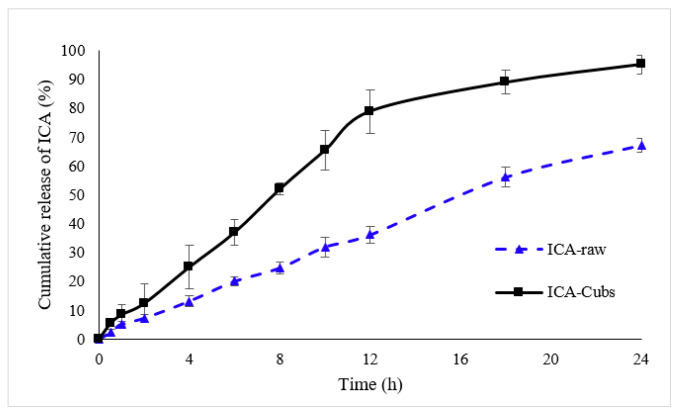
In vitro comparative release profiles of ICA-raw and ICA-Cubs in the phosphate buffer (pH 7.4). Data are expressed as the mean ± SD (n = 3).

Figure 7 shows the in vitro release profiles of the ICA in the PBS from the Cubs and the ICA-raw. From the data, it is evident that the release profile and extent of ICA release were enhanced by the delivery system of the ICA. The release profile of the ICA from the Cubs showed an initial burst release followed by a gradual release until 96.23 ± 3.231% was reached within the time frame of 24 h, whereas there was a slow and incomplete release of ICA (67.34 ± 2.424%) from the ICA-raw. 

### 3.3. Cell-Based Analysis Results of ICA-Cubs

#### 3.3.1. Evaluation of In Vitro Cytotoxicity 

**Figure 8:**
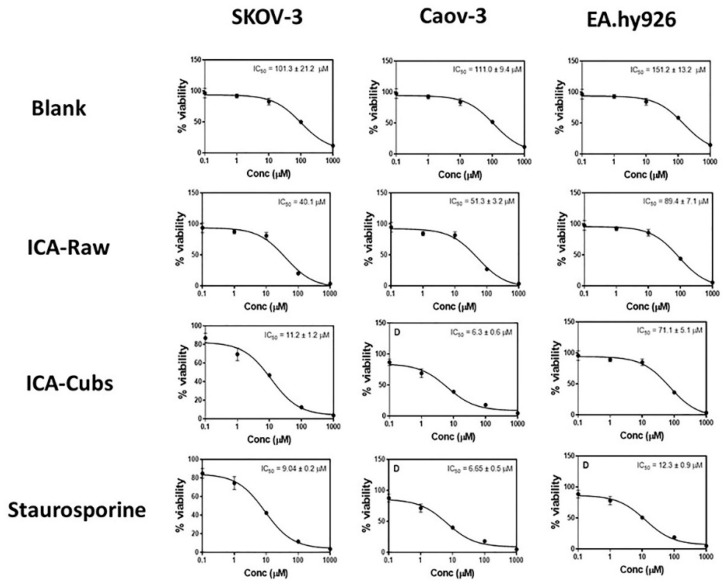
Dose response curves for the blank-Cubs, ICA-raw, ICA-Cubs and Staurosporine against the SKOV-3, Caov-3, and EA.hy926 cell lines. Data are expressed as the mean ± SD (n = 4 runs).

The cytotoxic profile of the free drug revealed a dose-dependent cytotoxic potential against the SKOV-3 cells. However, the IC_50_ value of the ICA-Cubs (11.2 ± 0.2 µM), when compared with that of the ICA-raw (40.1 ± 4.0 µM), indicates the potent cytotoxic role of the ICA-Cubs against the growth of the cancer cells. Our findings are depicted in Figure 8. Further, the optimized formula was examined in an addental ovarian cancer cells Caov 3. Blank-Cubs, ICA- raw, and ICA-Cubs showed IC_50_ of values 111.0 ± 9.4, 51.3 ± 3.2, 6.3 ± 0.6. All preparations exhibited IC_50_ vales greater than 30 µM against EA.hy926 non-cancerous endothelia cells.

**Figure 9:**
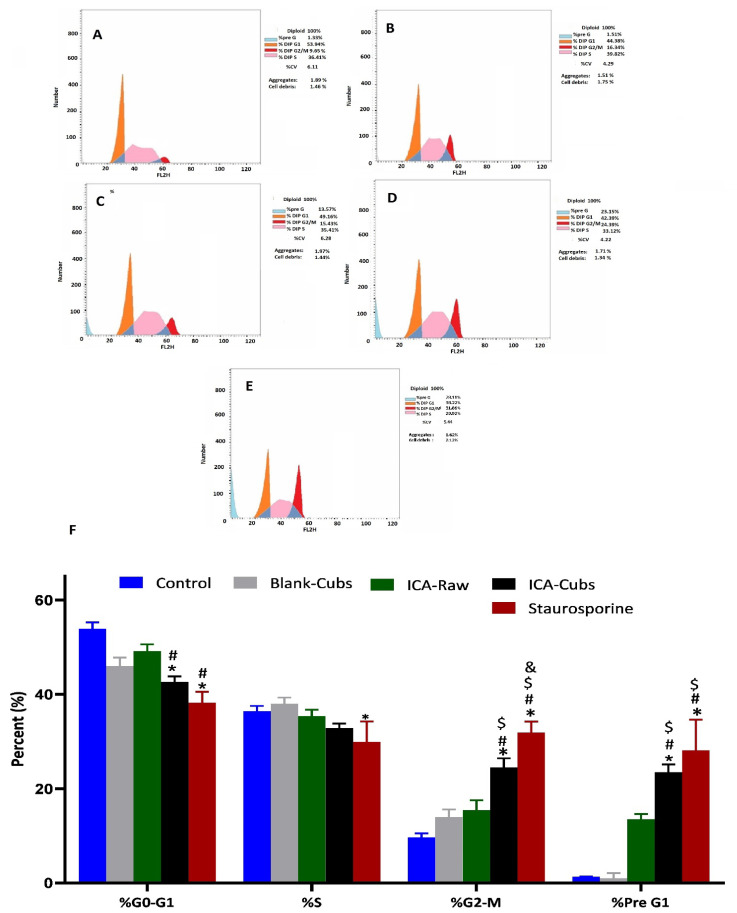
Flow cytometric analysis of the cell cycle arrest, (**A**) Skov-3 cells without treatment, then after treatment with (**B**) the blank-Cubs, (**B**) ICA-raw, (**C**) ICA-Cubs, (**D**)Staurosporine against the SKOV-3 cell line. (**E**) Bar diagram of the different cycle phases. Data are expressed in (**F**) as the mean ± SD (n = 4 runs). * represents a significant difference when compared with untreated cells (control) (*p* < 0.05), whereas # represents a significant difference when compared with the blank-Cubs (*p* < 0.05) and $ represents a significant difference when compared with the ICA-raw (*p* < 0.05) and & represents a significant difference when compared with the ICA-Cubs (*p* < 0.05).

The ICA-Cubs was found to inhibit the G0‒G1-phase and S-phase, where arrest occurred significantly compared with the control and the blank-Cubs. Similarly, relative cell cycle enhancement in the G2‒M-phase and pre-G1-phase was also observed significantly compared with the control, blank-Cubs, and ICA-Cubs, as shown in Figure 9. 

#### 3.3.2. Determination of Apoptotic Potential

**Figure 10:**
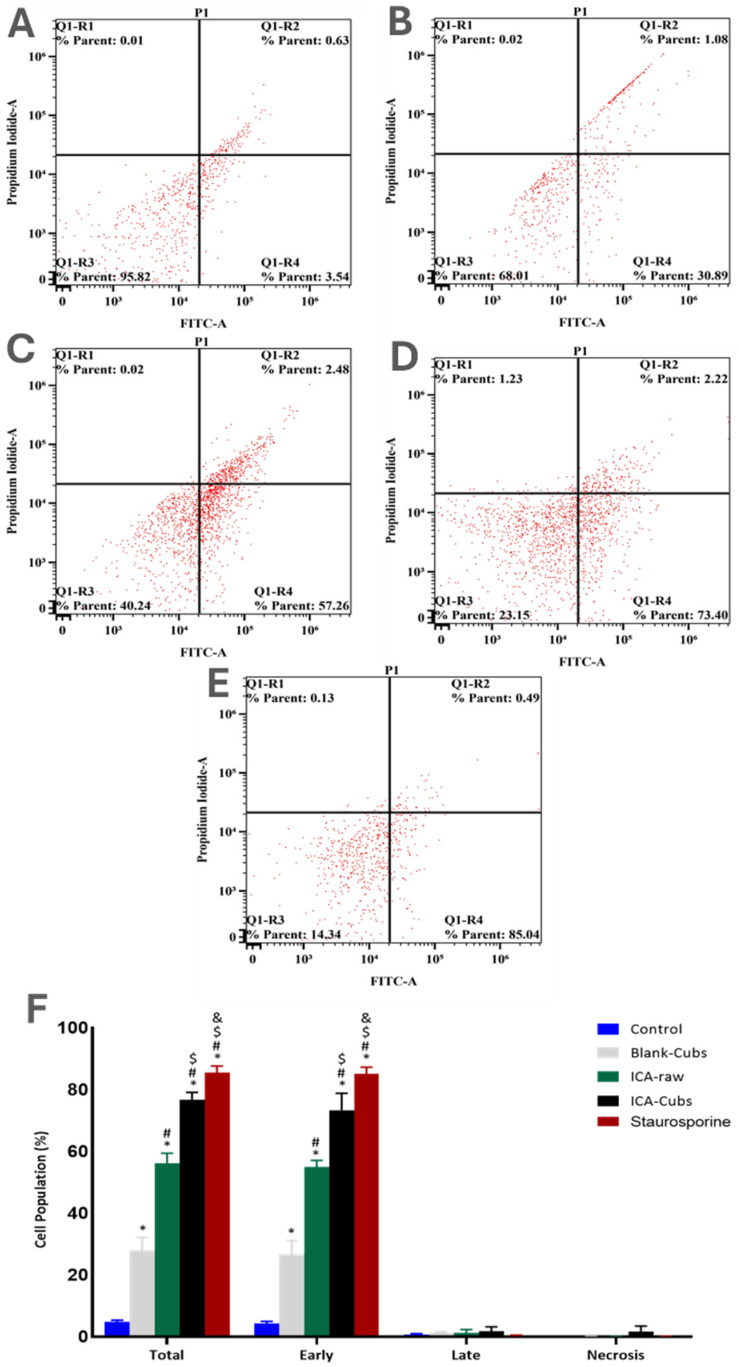
Apoptotic potential using flow cytometric analysis following Annexin V staining of the SKOV-3 cell line without treatments (**A**) and after treatment with (**B**) blank-Cubs, (**C**) ICA-raw, (**D**) ICA-Cubs and with (**E**) staurosporine. Data are expressed in (**F**) as the mean ± SD (*n* = 3 runs). Representation of SKOV-3 cell death following apoptotic and necrotic assay by cytometric analysis after annexin V staining. * represents a significant difference when compared with untreated cells (control) (*p* < 0.05), whereas # represents a significant difference when compared with the blank-Cubs (*p* < 0.05), $ represents a significant difference when compared with the ICA-raw (*p* < 0.05), and & represents a significant difference when compared with the ICA-Cubs (*p* < 0.05).

Similarly to the existing literature, our analysis using the Annexin V-FITC Apoptosis Kit revealed early and total apoptosis on the SKOV-3 cells when treated with the ICA-raw (Figure 10). Compared with the results of the ICA-raw, the ICA-Cubs induced greater and more distinctive early and total apoptosis. As we looked at necrosis, it was observed that the effect of the ICA-Cubs was not significantly greater than that of the ICA-raw group. 

#### 3.3.3. Measurement of Reactive Oxygen Species Production

**Figure 11:**
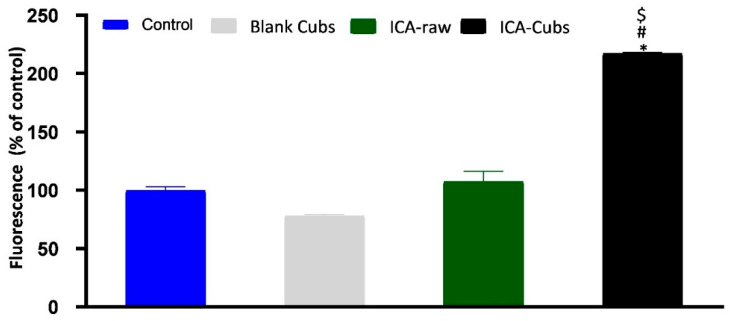
Generation of reactive oxygen species (ROS) in SKOV-3 cells following treatment with blank-Cubs, ICA-raw, and ICA-Cubs. Data are expressed as the mean ± SD (n = 4 runs). * represents a significant difference when compared with untreated cells (control) (*p* < 0.05), whereas # represents a significant difference when compared with the blank-Cubs (*p* < 0.05) and $ represents a significant difference when compared with the ICA-raw (*p* < 0.05).

The ROS generation by the cells treated with different samples is shown in Figure 11. The blank-Cubs was found to decrease the fluorescent level when compared with the control, but the reduction was clinically insignificant. Alternatively, treatment with the ICA-raw showed a significant increase when compared with the control and blank-Cubs treatment groups; however, treatment with the ICA-Cubs had the highest fluorescence with a significant difference when compared with the cells treated with the control, blank-Cubs, and ICA-raw. 

#### 3.3.4. Measurement of TNF-α and Nitric Oxide Production

**Figure 12:**
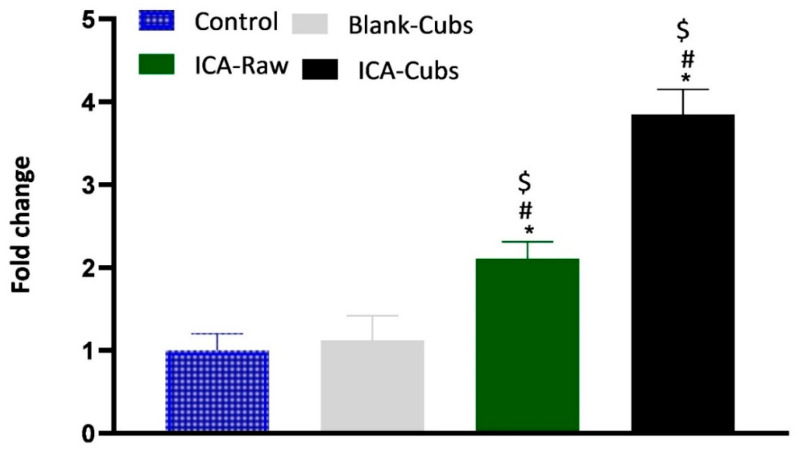
Decrease in the folds of TNF-α production in SKOV-3 cells following treatment with blank-Cubs, ICA-raw, and ICA-Cubs. Data are expressed as the mean ± SD (n = 4 runs). * represents a significant difference when compared with untreated cells (control) (*p* < 0.05), whereas # represents a significant difference when compared with the blank-Cubs (*p* < 0.05) and $ represents a significant difference when compared with the ICA-raw (*p* < 0.05).

Investigation of TNF-α in the present experiment was performed to determine the role of the formulation and the ICA-raw in the production of TNF-α within the SKOV-3 cell lines. As shown in Figure 12, treatment with ICA-raw and ICA-Cubs can significantly increase the production of TNF-α within ovarian cancer cells, where the efficacy of the ICA-Cubs is significantly higher when compared with the ICA-raw treatment. 

**Figure 13:**
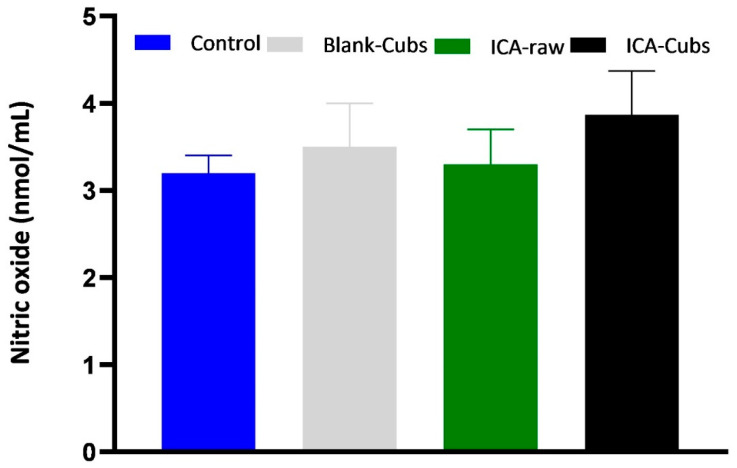
Generation of NO in SKOV-3 cells following treatment with blank-Cubs, ICA-raw, and ICA-Cubs. Data are expressed as the mean ± SD (n = 4 runs).

We used the kit-based assay to analyze the role of the NO inhibitory effect. Figure 13 depicts the effect of different formulations and ICA-raw in the generation of NO. From the findings it could be inferred that the generation of NO by the SKOV-3 cell line is decreased when the cells are incubated with ICA-Cubs; however, such a decrease in NO production is insignificant.

#### 3.3.5. Caspase-3 and p53 Assay

**Figure 14:**
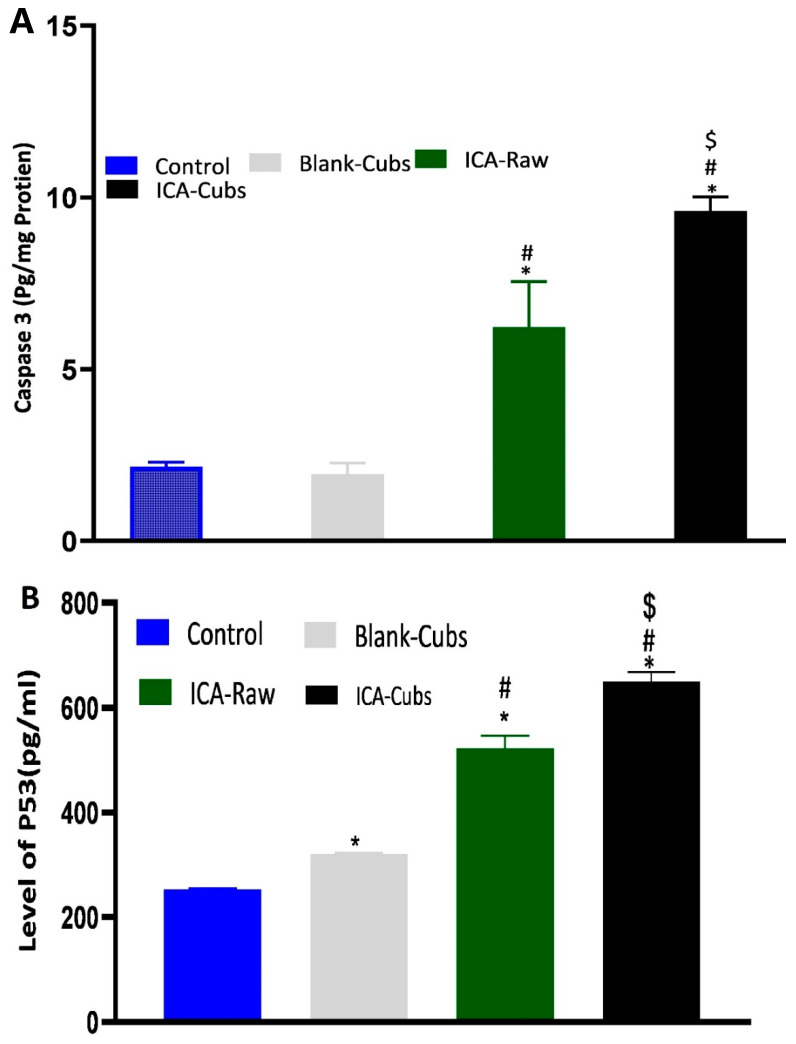
The expression of caspase-3 (**A**) and p53 (**B**) in SKOV-3 cells following incubation with blank-Cubs, ICA-raw, and ICA-Cubs. Data are expressed as the mean ± SD (n = 4 runs). * represents a significant difference when compared with the control (*p* < 0.05), whereas # represents a significant difference when compared with the blank-Cubs (*p* < 0.05) and $ represents a significant difference when compared with the ICA-raw (*p* < 0.05).

Determination of caspase-3 by the treatment of samples could be correlated with the apoptotic potential because caspase-3 controls the DNA fragmentation and damage of the cellular protein [35,36,37]. Findings of the caspase-3 assay are summarized in Figure 14A, where it can be clearly seen that the ICA-raw and ICA-Cubs had a significant effect on the overexpression of caspase-3. 

## 4. Discussion

The development of a highly twisted cubosomal structure containing a lipid bilayer was obtained by the disruption of the formed cubic gel phase of GMO. A placebo (ICA-free) and ICA-Cubs were formulated where the mechanical stirring of the system with P407 stabilized the system. The formed colloidal dispersion was devoid of any aggregates with a white–opaque appearance. The compositions of the different formulas of ICA-Cubs are shown in Table 1. The actual (experimental) and predicted (fitted) values for the particle sizes of ICA-Cubs are in close agreement, as shown in Table 1. The percentage error, which is the percentage difference for all the values of the actual and predicted particle sizes, is also found to be very low. The obtained positive coefficients of ICA and GMO reveal that the particle size will increase with increasing ICA and GMO concentrations in the formulations. In contrast, the negative coefficient of P407 indicates that the particle size will increase with decreasing P407 concentrations in the formulations. The obtained significant *p*-values indicate that the effects of the ICA, GMO, and P407 concentrations on the particle size of the Cubs formulation of ICA are evident. Similarly, the negative effect of P407 on the particle size in the Pareto chart (see Figure 2) is in accordance with the negative coefficient in Equation (1) for the model term B. The results reflected in the main effects plot (see Figure 3A) for the particle size are also in agreement with those in the Pareto chart (see Figure 2) and Equation (1). GMO had the most significant effect; a sharp increase in the particle size was observed when the concentration of GMO was increased in the formulation. Similarly, a sharp increase in the particle size is demonstrated in Figure 4 with the ICA concentration. An increase in the P407 concentration resulted in a decreasing particle size. However, the effect of the ICA or P407 concentration was not as influential as the effect of the GMO concentration on the particle size of the ICA-Cubs. An increase in the EE of the ICA-Cubs formulation with increasing ICA, GMO, and P407 concentrations was evident in the Pareto chart showing the effect of the independent variables on entrapment in Figure 4 by the plus sign. The increased EE with increasing ICA, GMO, and P407 concentrations might be explained by the increasing content of all three independent variables. Our findings are in agreement with those in the reported literature [23,24]. As per the Pareto chart, the P407 concentration had the greatest effect on the EE, and this is further supported by the main plot (see Figure 5A) and contour plot (see Figure 5B). The increased size of the ICA-Cubs during the dynamic light-scattering technique might be explained by the hydrodynamic diameter of the dispersed system in the aqueous environment [38]. Higher values of the surface charge indicating superior stability of the dispersed system might contribute to the repulsion of the charged vesicles and thereby prevent the van der Waals force from causing the directed aggregation of the dispersed system [39]. Further analysis of the morphology revealed that the Cubs were uniform and polyangular in shape with a smooth surface and a scattered distribution. This scattering of the vesicles could be attributed to the high surface charge. Cubs could help in releasing the drug from the formulation in a controlled manner, as depicted in the in vitro release profile. Superior release of the entrapped drug was found to increase the cytotoxic potential of the ICA-loaded formulation in a dose-dependent manner [28]. It can be clearly stated that the lowest IC_50_ value of the ICA-Cubs (11.2 µM), compared with that of the ICA-raw (40.1 ± 4.0 µM), could be attributed to the increased cellular permeability of ICA when delivered via the cubosomal delivery approach compared with that of the ICA-raw [40]. Analysis of the cell cycle arrest by the formulations was measured by quantification of the DNA content using flow cytometry analysis. Thus, the IC_50_ concentrations of the ICA-Cubs and the analysis of the ICA-mediated arrest of the cell cycle are presented in Figure 9. The analysis showed that there was a significant difference in all the different phases of the cell cycles.

Our results for the ICA-Cubs against the SKOV-3 cell line are comparable to those in the published literature, and the arrests of the different phases are also comparable [10]. Apoptosis is an important step in different biological processes; it can be stimulated by many things and lead to various morphological changes [32,41]. The literature suggests that the ICA can promote early and late apoptosis when compared with the control. This apoptotic potential of ICA is much greater when it is treating cancer cells [11]. This improved efficacy could be explained by the potential of increased solubility with the controlled and complete release of ICA over time in the developed cubosomal approach. Nowadays, dichloro-dihydro-fluorescein diacetate is widely used in cell-based assays to detect the antioxidant potential of the components; the characteristics of this chemical that produce a highly fluorescent end product upon oxidation are utilized [42]. This diacetate compound can cross the cell membrane and be cleaved to dichloro-dihydro-fluorescein by the cellular esterase without affecting the cellular viability [42,43]. Findings of our treatments on the generation of ROS are in agreement with those of the previously published literature [10]. This increased generation of ROS might be correlated with the elevated levels of the apoptotic potential of the ICA [43], particularly when it is delivered using the cubosomal delivery approach. Thus, it is expected that treatment with ICA-Cubs has a greater potential to reduce the production of TNF-α within the cytosol of the SKOV-3 cells, and this could interfere with angiogenesis in the tumor microenvironment and the growth, proliferation, invasion, and metastasis of cancer cells [44]. Similar results on the enhancement of caspase-3 efficacy using ICA had been reported in the literature [45,46]. Furthermore, the effect of the formulation approach is much higher because the production of caspase-3 by ICA-Cubs is significantly higher than by ICA-raw. This might be due to the increased internalization of ICA in the novel formulation; the placebo formulation did not induce any efficacy in the caspase-3 content in the ovarian cancer cells. This role of caspase-3 in the treatment group might be correlated with the p53 expression, which might affect cell growth and the metastasis of cancer cells. The corresponding expression of p53 in the SKOV-3 cell line is presented in Figure 14B, showing a significant increase in the level of p53 when treated with ICA and ICA-Cubs, similar to that of caspase-3. P53 is an important tumor suppressor, and its overexpression leads to arrest of the cell cycle and promotion of the apoptosis of cancer cells. Thus, we could speculate that the overexpression of p53 might be involved in the anti-cancer potential of ICA [13], where cubosomal delivery has a potential role in improving efficacy by improving the cellular availability of ICA.

## 5. Conclusions

In summary, ICA-Cubs using GMO and P407 was successfully developed through optimization by the Box‒Behnken statistical design. The optimized vesicles were cubic in shape with an EE of 98.8%. The controlled release pattern of ICA-Cubs was delayed compared with that of ICA-raw over 24 h. The vesicular delivery approach might increase the cellular uptake of ICA; its cytotoxicity towards the ovarian cancer cells was decreased by one-fifth over that of ICA-raw, and it had a safe profile in normal cells. There was increased generation of ROS by 2-fold and overexpression of p53 and caspase-3 by 1.4-fold in the SKOV-3 cell line. The decreased growth and arrest in the cell cycle might be due to the upregulation of TNF-α by 1.8-fold. Overall, the cubosomal formulation approach for the ICA could be used as an effective delivery tool for improved efficacy against ovarian cancer cells; however, further studies are recommended to explore and establish the safety and efficacy in in vivo models.

## Figures and Tables

**Table 1:** Experimental runs according to the Box–Behnken statistical design: The independent variables and dependent variables with the levels of the independent variables, ICA concentration, GMO concentration. and P 407 concentration.

Run	Values of Independent Variables		Dependent Variable (Y)				
	ICA Concentration (mg/g)	GMO Concentration (g/g)	P407 Concentration (mg/g)	Particle Size (nm)	Entrapment Efficiency (%)		
				Actual	Predicted	Actual	Predicted
1	100	0.80	78.00	103.2 ± 1.21	104.67	91.8 ± 1.50	91.93
2	100	0.60	78.00	99.5 ± 3.15	94.25	78.3 ± 2.11	78.00
3	150	0.80	78.00	112.2 ± 4.86	112.00	97.9 ± 5.32	98.71
4	50	0.60	78.00	141.6 ± 3.71	139.75	88.9 ± 2.25	90.34
5	150	0.80	78.00	117.7 ± 4.38	112.00	97.3 ± 3.22	98.71
6	100	0.80	31.00	105.1 ± 4.12	104.67	91.9 ± 3.31	91.93
7	50	0.80	78.00	108.3 ± 4.41	113.00	80.8 ± 1.11	79.39
8	100	1.00	78.00	183.6 ± 6.37	179.25	83.1 ± 4.42	83.08
9	150	0.80	31.00	179.2 ± 2.12	179.25	96.9 ± 2.25	95.19
10	50	0.80	125.00	97.3 ± 4.25	93.50	96.1 ± 2.81	94.36
11	100	0.80	78.00	106.5 ± 3.21	104.67	92.1 ± 3.34	91.93
12	150	1.00	31.00	88.2 ± 2.22	89.25	90.3 ± 0.62	88.86
13	100	1.00	78.00	73.1 ± 2.15	76.75	94.3 ± 0.87	94.33
14	100	0.80	125.00	139.5 ± 3.24	143.75	98.5 ± 1.10	98.80
15	50	0.60	31.00	78.0	77.75	85.4	87.11
16	100	1.00	125.00	103.0	104.67	91.8	91.93
17	100	0.60	125.00	99.0	94.25	78.3	78.00
Independent variable		Levels					
		Low (−1)	Medium (0)		High (1)		
ICA concentration (mg/g)		50	100		150		
GMO concentration (g/g)		0.6	0.8		1.0		
P407 concentration (mg/g)		31	78		125		

ICA, icariin; GMO, glyceryl monooleate; P407, poloxamer 407. Dependent variables: Y1 = particle size; Y2 = entrapment efficiency.

**Table 2:** Analysis of variance data for particle size.

Source	Sum of Squares	F-Ratio	*p*-Value
A:ICA	1300.5	41.77	0.0013
B:GMO	11,552.0	371.05	0.0000
C:P407	1404.5	45.11	0.0011
AA	80.4103	2.58	0.1689
AB	196.0	6.30	0.0539
AC	49.0	1.57	0.2651
BB	546.564	17.56	0.0086
BC	81.0	2.60	0.1677
CC	164.103	5.27	0.0701
Total error	155.667		
Total (corr.)	15,448.4		

**Table 3:** Analysis of variance data for entrapment efficiency.

Source	Sum of Squares	F-Ratio	*p*-Value
A:ICA	21.78	5.38	0.0682
B:GMO	45.6013	11.26	0.0202
C:P407	513.601	126.79	0.0001
AA	2.3141	0.57	0.4838
AB	2.4025	0.59	0.4760
AC	1.1025	0.27	0.6242
BB	2.17026	0.54	0.4970
BC	0.09	0.02	0.8873
CC	25.281	6.24	0.0546
Total error	20.2542		
Total (corr.)	632.429		

## Data Availability

Data sharing not applicable. No new data were created or analyzed in this study. Data sharing is not applicable to this article.
