# Correction: Fahmy et al. Optimized Icariin Cubosomes Exhibit Augmented Cytotoxicity against SKOV-3 Ovarian Cancer Cells. *Pharmaceutics* 2021, *13*, 20

**DOI:** 10.3390/pharmaceutics17020145

**Published:** 2025-01-22

**Authors:** Usama A. Fahmy, Omar Fahmy, Nabil A. Alhakamy

**Affiliations:** 1 Department of Pharmaceutics, Faculty of Pharmacy, King Abdulaziz University, Jeddah 21589, Saudi Arabia; nalhakamy@kau.edu.sa; 2 Center of Excellence for Drug Research & Pharmaceutical Industries, King Abdulaziz University, Jeddah 21589, Saudi Arabia; 3 Department of Urology, University Putra Malaysia (UPM), Selangor 43400, Malaysia; docomar82@gmail.com; 4 Department of Urology, University Hospital of Tübingen, Eberhard-Karls University, 72076 Tübingen, Germany

## Error in Figure

In the original publication [1], an error in Figure 10 was identified. The flow cytometry experiment was repeated to ensure data accuracy. The corrected version of Figure 10 is presented below, along with minor changes in Section 3.3.2. Determination of Apoptotic Potential.

## Text Correction

In the description of “Section 2.5.3. Cell Cycle Analysis”, a minor modification is requested as follows:

Finally, the stages of cells in different cell cycles were determined using a Cytek^®^ Northern Lights 2000 spectral flow cytometer (Cytek Biosciences, Fremont, CA, USA), and the acquired data were analyzed by using SpectroFlo™ Software version 2.2.0.3 (Cytek Biosciences, Fremont, CA, USA).

In the description of the results in “Section 3.3.2. Determination of Apoptotic Potential”, a minor modification is requested as follows:

Similarly to the existing literature, our analysis using the Annexin V-FITC Apoptosis Kit revealed early and total apoptosis on the SKOV-3 cells when treated with the ICA-raw (Figure 10). Compared with the results of the ICA-raw, the ICA-Cubs induced greater and more distinctive early and total apoptosis. As we looked at necrosis, it was observed that the effect of the ICA-Cubs was not significantly greater than that of the ICA-raw group.

The authors state that the scientific conclusions are unaffected. This correction was approved by the Academic Editor. The original publication has also been updated.

## Figures and Tables

**Figure 10:**
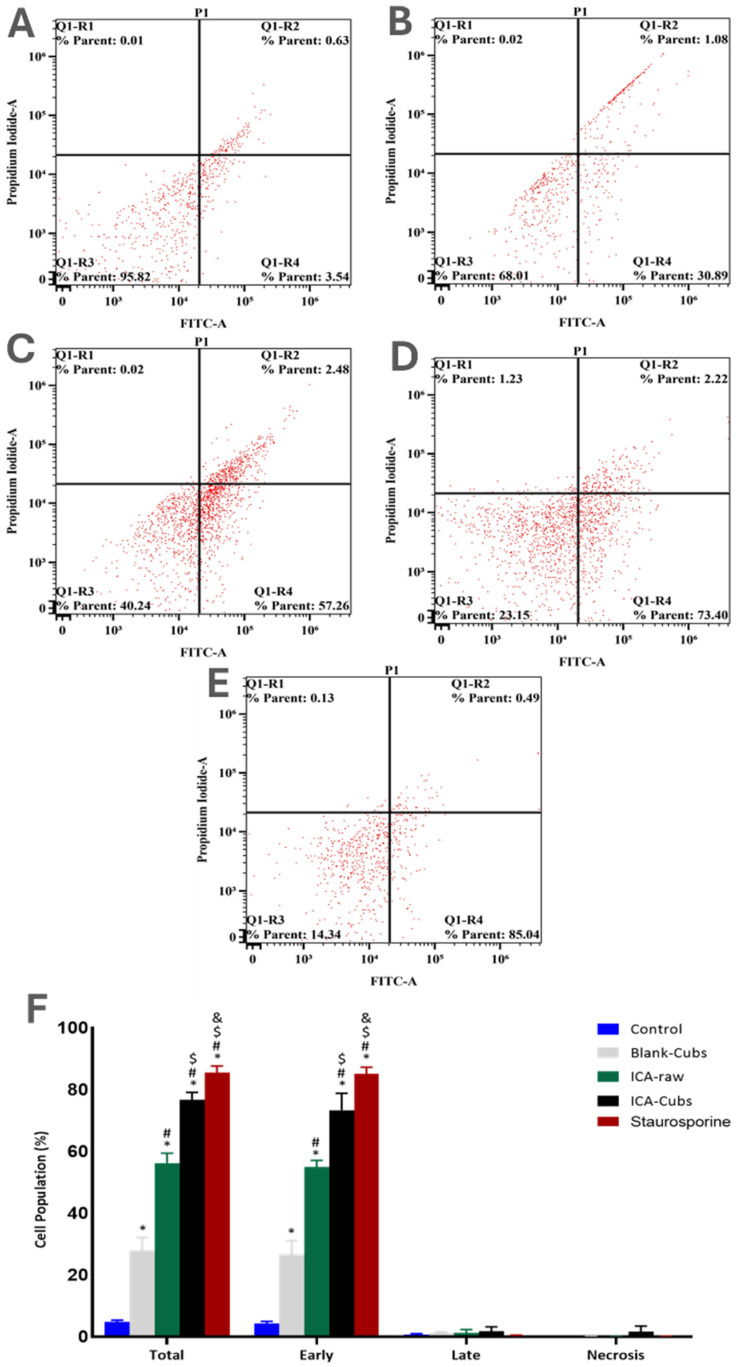
Apoptotic potential using flow cytometric analysis following Annexin V staining of the SKOV-3 cell line without treatments (**A**) and after treatment with (**B**) blank-Cubs, (**C**) ICA-raw, (**D**) ICA-Cubs and with (**E**) staurosporine. Data are expressed in (**F**) as the mean ± SD (*n* = 3 runs). Representation of SKOV-3 cell death following apoptotic and necrotic assay by cytometric analysis after annexin V staining. * represents a significant difference when compared with untreated cells (control) (*p* < 0.05), whereas # represents a significant difference when compared with the blank-Cubs (*p* < 0.05), $ represents a significant difference when compared with the ICA-raw (*p* < 0.05), and & represents a significant difference when compared with the ICA-Cubs (*p* < 0.05).
